# From classic to cutting-edge: technological approaches to respiratory physiological signals in assessing sleep-disordered breathing

**DOI:** 10.1007/s44470-026-00088-6

**Published:** 2026-05-26

**Authors:** Ambrose A. Chiang, Joyce Lee-Iannotti, Brennan Torstrick, Richard B. Berry, Nancy A. Collop

**Affiliations:** 1 Department of Medicine, Case Western Reserve University, Cleveland, OH USA; 2 Division of Sleep Medicine, Louis Stokes Cleveland VA Medical Center, 10701 East Blvd., Cleveland, OH 44106 USA; 3 Division of Pulmonary, Critical Care, and Sleep Medicine, University Hospitals Cleveland Medical Center, Cleveland, OH USA; 4 Barrow Neurological Institute, Phoenix, AZ USA; 5 University of Arizona College of Medicine, Phoenix, AZ USA; 6 Huxley Medical, Atlanta, GA USA; 7 University of Florida, Gainesville, FL USA; 8 Emory University, Atlanta, GA USA

**Keywords:** Polysomnography, Home sleep apnea testing, Wearable, Sleep technology, Photoplethysmography, Peripheral arterial tonometry

## Abstract

**Abstract:**

The diagnostic armamentarium for sleep-disordered breathing (SDB) has undergone substantial expansion in recent years, alongside the heightened awareness of SDB, its systemic impact on health, and the proliferation of home sleep apnea testing (HSAT). Many contemporary diagnostic modalities incorporate physiological measurement techniques that draw upon concepts primarily investigated in research paradigms, but may not align with existing HSAT classification frameworks, particularly regarding respiratory signal acquisition and analysis. This narrative review synthesizes current respiratory analysis techniques employed in SDB diagnosis, categorizing them broadly into three principal measurement domains: airflow dynamics, respiratory effort mechanics, and sympathetic activation patterns. Emphasis is placed on the historical background, underlying physiological mechanism, and clinical utility, while evaluating the methodological strengths and inherent limitations of each measurement approach.

**Brief summary:**

While prior literature reviews have extensively documented conventional respiratory analysis methodologies in sleep apnea diagnostics, the rapidly evolving landscape of cutting-edge diagnostic technologies and their physiological signals remain insufficiently addressed in the current literature. This respiratory technology-centered narrative review bridges this crucial knowledge gap through a rigorous and comprehensive examination of both established and emerging respiratory assessment modalities. Furthermore, we posit that the existing sleep testing classification frameworks no longer adequately reflect the breadth and sophistication of contemporary diagnostic technologies, and that their revision represents a pressing imperative for the sleep medicine community. We advocate a reconceptualization of sleep testing taxonomies to ensure clinical relevance and diagnostic utility amid accelerating technological evolution.

## Introduction

Sleep-disordered breathing (SDB) represents a global health burden affecting nearly one billion adults worldwide, conferring substantially elevated risk of cardiovascular pathologies, cerebrovascular diseases, and mortality [1, 2]. Timely diagnosis and therapeutic intervention are critical in mitigating comorbid complications and enhancing quality of life [3]. Despite this clinical imperative, epidemiological data suggest that as many as 80% of patients with SDB remain undiagnosed, representing a significant diagnostic gap [4].

As SDB awareness builds among both the general population and referring clinicians, the unprecedented influx of undiagnosed patients places substantial resource strain on existing sleep medicine facilities. Conventional diagnostic paradigms, such as in-lab polysomnography (PSG) and home sleep apnea testing (HSAT), measuring nasal airflow and respiratory effort, demonstrate inherent limitations characterized by operational complexity, reduced patient tolerability, and elevated technical failure rates [5, 6]. Consequently, novel sleep apnea diagnostic platforms have emerged to optimize usability and expand diagnostic capacity [7, 8]. While these cutting-edge sleep technologies have been investigated in research settings, they may not always conform to established classification frameworks for SDB evaluation [9, 10].

**Fig. 1:**
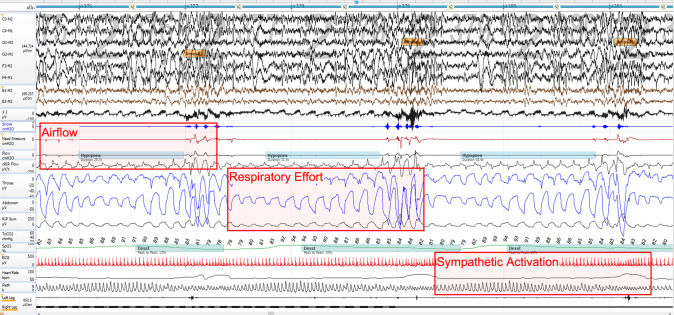
Standard polysomnography montage highlighting the three respiratory analysis signal domains

To achieve clinical utility, any clinically viable diagnostic modality must yield sufficient clinical information to confidently assess SDB while complying with the guidelines from professional medical societies and payer reimbursement policies. At a minimum, physiological metrics including respiration, oxygen saturation, and cardiac rate must be captured. These parameters collectively characterize typical SDB events, which progress from respiratory disturbance to oxygen desaturation and/or terminate in sympathetically mediated arousals (“autonomic arousals”) (Fig. 1).

While oximetry and cardiac rate monitoring methodologies are better defined, respiratory signal acquisition and analysis methods remain complex due to the pervasive influence of respiration in multiple physiological systems. Overall, respiratory monitoring can be broadly categorized into three principal domains: respiratory airflow dynamics, respiratory effort mechanics, and sympathetic activation patterns [11, 12]. Each domain can be assessed using various respiratory analysis approaches with varying levels of technical complexity and signal fidelity. Tables 1, 2, 3 summarize the three respiratory signal monitoring domains, specific measurement methodologies, and their respective strengths and limitations.

**Table 1:** Summary of airflow measurement methods

Measurement methodology	Strengths	Limitations
Pneumotachograph	- Gold standard for airflow - Accurate and linear volumetric flow	- Obtrusive, uncomfortable mask - Technically complex, clinically impractical
Thermistor	- Inexpensive, widespread - Confirms airflow presence/absence	- Limited hypopnea detection. Does not quantify airflow - Susceptible to displacement and room drafts
Nasal pressure transducer	- Simple, inexpensive, widespread - High hypopnea sensitivity	- Less accurate during mouth breathing - Overestimates apneas - Susceptible to displacement, nasal secretion obstruction
Thoracoabdominal effort-derived flow	- Simple, inexpensive, widespread - Comfortable and non-obtrusive	- Belts are susceptible to displacement, rolling, folding - Often requires calibration; difficult in home setting
Tracheal sounds	- Captures total oronasal flow - Comfortable, less prone to displacement	- Hypopnea detection can be challenging - Anatomical misalignment with loose skin on neck

**Table 2:** Summary of respiratory effort measurement methods

Measurement methodology	Strengths	Limitations
Esophageal pressure	- Gold standard for respiratory effort - Reflects intrathoracic pressure changes	- Invasive, uncomfortable, technically complex - Prone to signal quality challenges and cardiac artifact
Chest wall electromyography (EMG)	- Directly measures neural respiratory drive - Detects feeble respiratory efforts	- Technically complex setup - Prone to signal quality issues and ECG interference
Respiratory inductance plethysmography (RIP)	- Simple, non-obtrusive, widespread - Detects paradoxical breathing	- Susceptible to displacement, rolling, folding - May over detect centrals during feeble effort or in obese
Chest/Tracheosternal movement	- Simple and non-obtrusive - Less prone to displacement	- Signal quality can depend on body position and sensor placement location
Respiratory-induced pulse wave modulation	- Simple and non-obtrusive - Systemic signal less affected by posture	- Optical signal quality influenced by perfusion, skin pigmentation, hair, nail polish, tattoos, and motion
Pulse transit time/Pulse arrival time	- Reflects intrathoracic pressure changes - Systemic measure less dependent on position	- Confounded by autonomic instability (e.g., in REM) - Requires two synchronized sensors (e.g., ECG and PPG)
Suprasternal pressure	- Simple, less prone to displacement - Correlates well with esophageal pressure	- Requires airtight seal - Anatomical misalignment with loose skin on neck
Mandibular motion	- Non-obtrusive - Correlates well with esophageal pressure	- Facial hair may require shaving if present - Restricting head against pillow may impact signal quality

*ECG*, electrocardiography; *EMG*, electromyography; *PPG*, photoplethysmography; *REM*, rapid eye movement; *RIP*, respiratory inductance plethysmography

**Table 3:** Summary of sympathetic activation measurement methods

Measurement methodology	Strengths	Limitations
Microneurography	- Gold standard for sympathetic activation - Direct MSNA measurement	- Invasive and technically complex, possible complications - Limited to research settings
Cardiac rate response	- Well-known autonomic SDB response - Easily measured with ECG or PPG	- Limited specificity confounded by autonomic instability - Impacted by medications, pacemakers, arrhythmias
Cardiopulmonary coupling	- Simple and convenient, using ECG or PPG - Improves specificity over cardiac rate alone	- Less intuitive to interpret frequency domain signals - May detect central events with further validation needed
Peripheral vasoconstriction	- Sensitive to autonomic arousals - PWA response associated with CV risk	- Limited specificity confounded by autonomic instability - May be limited for certain anatomies
Pulse transit time/Pulse arrival time	- Sensitive to autonomic arousals - PTT response associated with CV risk	- Limited specificity confounded by autonomic instability - Influenced by vascular compliance

*CV*, cardiovascular; *ECG*, electrocardiography; *MSNA*, muscle sympathetic nerve activity; *PPG*, photoplethysmography; *PTT*, pulse transit time; *PWA*, pulse wave amplitude; *SDB*, sleep-disordered breathing

While past literature reviews have comprehensively documented conventional respiratory analysis methodologies, contemporary literature lacks coverage of recent cutting-edge diagnostic technologies [13–17]. This respiratory signal technology-centered narrative review addresses this knowledge gap by providing an integrated analysis of both established and emerging respiratory assessment modalities, critically evaluating their diagnostic utility and practical limitations. Additionally, we examine how these evolving techniques may streamline SDB diagnostic pathways, particularly in real-world at-home settings.

## Respiratory analysis

### Airflow measurement techniques in sleep-disordered breathing

Airflow measurement constitutes a primary conventional physiological parameter in SDB respiratory monitoring, where airway occlusion directly manifests as alterations in ventilatory flow. Quantitative airflow metrics have served as a fundamental signal for SDB evaluation for decades, enabling respiratory event classification based on the magnitude of airflow reduction (e.g., ≥ 90% airflow drop for apneas, ≥ 30% drop for hypopneas) [18].

#### Pneumotachograph

Pneumotachography represents the gold standard for direct airflow measurement, utilizing differential pressure transduction across a calibrated resistive element [13, 16, 19]. This technique relies on a tightly sealed face mask interfaced with a pressure sensor to quantify ventilatory airflow, exploiting a near-linear pressure-flow relationship. While providing accurate volumetric flow, the pneumotachograph is rarely employed in clinical practice, particularly in home settings, due to its obtrusive nature, technical complexity, and economic constraints. The limitations of pneumotachography prompted the development of alternative airflow detection technologies. More convenient and practical surrogates of airflow were introduced in the 1980s and gained wide adoption with the rise of home sleep apnea testing in the early 2000s. Today, the most common airflow surrogates are thermistors and nasal pressure transducers.

#### Thermistors

Thermistors are positioned in proximity to the nasal and oral regions to detect temperature differentials between exhaled warm air and inhaled ambient cooler air. Due to their inherent slow and non-linear thermal response, traditional thermistors can only confirm the presence of airflow but cannot accurately quantify its magnitude. As a result, thermistors can only reliably detect apneas but are limited in their ability to identify hypopneas [13, 16]. Alternative thermal-based sensors using polyvinylidene fluoride (PVDF) exhibit a faster, more linear response relative to thermistor oronasal flow, demonstrating superior agreement with the pneumotachograph in detecting both apneas and hypopneas [20, 21].

While more comfortable than the masked pneumotachograph, thermistors can be difficult to tolerate for some patients and are prone to displacement during the night [5, 22]. Furthermore, environmental interference from airflow convection, such as room air drafts, can compromise signal integrity, potentially impacting data reliability [23]. Novel wearables with non-traditional placement of the thermistor sensor on the chin have recently been introduced and warrant further characterization [8, 24].

#### Nasal pressure transducers

Nasal pressure transducers quantify airflow by detecting dynamic pressure variations inside a nasal cannula during respiration [13]. This methodology provides superior sensitivity in hypopnea detection compared to conventional thermistors, attributable to its enhanced resolution of subtle flow limitations. A square root transformation is frequently applied to the measured pressure signal to correlate with airflow more linearly [18]. However, a critical limitation of nasal pressure transducers is their contingency upon nasal breathing, rendering them more susceptible to signal loss in mouth-breathing individuals during sleep [14]. Some oronasal pressure transducers are available that mitigate this issue by adding an extension from the nasal prongs to capture oral airflow. Like thermistors, nasal pressure transducer cannulas can also be uncomfortable for patients and prone to displacement during the night. Cannula prongs can also be obstructed by accumulated nasal secretions, further affecting accuracy. To mitigate these limitations, contemporary PSG systems often employ a dual-sensor configuration, integrating both nasal pressure transducers and thermistors.

#### Thoracoabdominal effort-derived airflow

When thermistors and nasal pressure transducers yield suboptimal signals, dual respiratory inductance plethysmography (RIP) belts are recommended as an alternative airflow surrogate [18]. RIP belts, composed of elastic fabric embedded with sinusoidal wire sensing elements, are placed circumferentially around the thorax and abdomen to measure changes in their enclosed cross-sectional area during breathing. By assuming constant volume changes across both compartments of the torso, RIP belts can be used to estimate tidal volume using their sum (e.g., RIPsum) and airflow using the first derivative of their sum (e.g., RIPflow) [13, 25].

Notably, RIP signal fidelity is susceptible to degradation from belt displacement, folding, or positional shifts during sleep, particularly in obese individuals or pregnant women [6, 26]. These constraints have motivated the development of novel implementations leveraging the same concept of deriving airflow from the independent movement of the thorax and abdomen, via alternative sensing modalities: adhesive patches incorporating optical or motion sensors instead of elastic belts (see the “Chest/tracheo-sternal movement” section) and a simplified single thoracic belt system with optimized positioning at the costal margin [27–29]. Such effects can be mitigated with calibration, though this can be difficult to conduct in a home setting and may be impacted by belt displacement unless software-based continuous auto-calibration is implemented [16, 30].

#### Tracheal sounds

First investigated as early as 1980, tracheal breathing sounds provide an alternative airflow surrogate [23, 31]. These sensors are placed on the anterior neck near the suprasternal notch, where they derive airflow by measuring sound waves produced by air movement within the trachea. Tracheal sound signal processing algorithms can isolate the higher frequency bands representing airflow signals (typically 200–2000 Hz) from lower-frequency components, such as snoring (20–200 Hz) [26, 32]. Notably, tracheal sound-based wearables differ fundamentally from snoring-based devices in both placement and function. The tracheal location enables capture of total oronasal ventilation, circumventing the nasal breathing dependency of pressure transducers during oral breathing. While capable of distinguishing apneas from hypopneas, their ability to detect hypopneas may be limited [30, 33–35]. Coupling tracheal sounds with trachea-sternal motion using accelerometry may also improve the robustness of airflow estimation against confounding variables during sleep, such as changes in body position [32].

Unlike nasal cannulas and belt-based sensors, tracheal sound sensors are less prone to displacement throughout the night, though loose skin around the neck may limit their use or preclude their utilization altogether in some patients. Recent advancements have introduced wireless tracheal acoustic sensors, potentially enhancing patient comfort compared to traditional airflow monitoring devices [36, 37].

### Respiratory effort assessment in sleep-disordered breathing

Respiratory effort represents the physiological neuromuscular activation of the respiratory pump, encompassing diaphragmatic and intercostal muscles to generate negative intrathoracic pressure necessary to facilitate ventilation. In the context of SDB, respiratory effort patterns provide critical diagnostic information: preserved effort characterizes obstructive events, absent effort defines central events, and hybrid patterns distinguish mixed events. This differentiation forms the foundation for accurate pathophysiological classification and therapeutic strategy selection.

#### Esophageal pressure

Esophageal pressure monitoring constitutes the reference standard for respiratory effort quantification, providing a direct approximation of intrathoracic pressure [13, 16, 38]. This technique involves transnasal placement of either a fluid-filled balloon or transducer-tipped catheter into the distal esophagus. Despite its accuracy in capturing inspiratory pressure excursions, clinical utilization remains limited due to its invasive nature and patient discomfort. Additional technical challenges include cardiac contraction artifact and potential alterations in upper airway dynamics, necessitating catheter repositioning, with up to one-third of recordings yielding uninterpretable data [39–41].

#### Chest wall electromyography

Intercostal and diaphragmatic electromyography (EMG) provides direct quantification of neural respiratory drive and was endorsed as an alternative respiratory effort sensor in the initial American Academy of Sleep Medicine (AASM) scoring manual [42, 43]. Intercostal EMG is typically recorded in an intercostal space near the sternum, whereas diaphragmatic EMG conventionally utilizes the seventh and eighth intercostal spaces along the anterior axillary line. An optimized configuration targeting the eighth intercostal space at the right mid-axillary line enables dual-muscle assessment [43]. Diaphragmatic EMG amplitude demonstrates proportional increases with progressively negative esophageal pressure throughout obstructive events, reflecting escalating respiratory drive against airway obstruction [42]. Comparative studies indicate good concordance between chest wall EMG and respiratory inductance plethysmography (RIP) belts in classifying central, obstructive, and mixed events [44, 45].

Subtle respiratory efforts can be more apparent on chest wall EMG compared to RIP belts, potentially reducing over-detection of central events. However, electrocardiographic artifacts can obscure subtle inspiratory efforts, even with optimized right-sided electrode placement [43]. The primary downside of chest wall EMG is the requirement for a special setup and susceptibility to artifacts, particularly in obese patients, where increased soft tissue thickness and indistinct anatomical landmarks compromise signal quality [40]. Nearly a fourth of EMG-based respiratory effort recordings may be technically inadequate, constraining routine clinical implementation [43].

#### Respiratory inductance plethysmography belts

RIP has emerged as the predominant method for assessing respiratory effort in contemporary clinical sleep medicine practices. RIP systems employ elastic fabric bands containing sinusoidal conductive wire elements to measure thoracoabdominal circumferential excursions during breathing. While RIP demonstrates sufficient performance to differentiate obstructive and central events in routine clinical application, the correlation of the magnitude of thoracoabdominal excursions with gold-standard esophageal pressure may deviate from proportionality, particularly in obese patients or during periods of feeble respiratory effort, where RIP may falsely classify obstructive events as central [15, 40, 46, 47].

RIP belts offer substantially improved patient tolerability and operational simplicity compared to esophageal manometry, but reliable measurements depend critically upon accurate placement, which can be compromised by nocturnal belt movements and is particularly challenging in obese or pregnant populations [26, 35, 48]. Reported failure rates for technician-applied RIP belts range from 7 to 21%, with anticipated higher failure rates for patient-applied belts [49]. The concurrent use of a dual-belt configuration (thoracic and abdominal) offers measurement redundancy that can compensate for potential single-belt failure. This configuration also enables the derivation of surrogate airflow signals (see the “Thoracoabdominal effort-derived airflow” section) and enhances diagnostic capability through the detection of paradoxical breathing during obstructive events. Alternative respiratory effort belts employing strain gauges or piezoelectric sensors are generally not recommended, as they typically measure single-point tension rather than global circumferential change, potentially misrepresenting the overall respiratory efforts [47].

Beyond primary respiratory effort quantification, emerging applications of machine learning algorithms to RIP signals demonstrate potential for arousal detection and sleep stage classification, suggesting expanded diagnostic utility [50].

#### Chest/tracheo-sternal movement

Accelerometric quantification of thoracoabdominal displacement during respiration provides an alternative approach to respiratory effort assessment [51–54]. These sensors can capture the physical displacement of the chest wall as the chest expands and contracts. When affixed directly to the skin, accelerometer-based systems offer potential advantages over RIP, including enhanced patient comfort, improved convenience, and reduced susceptibility to nocturnal displacement. Accelerometric chest movement signals have demonstrated utility in differentiating obstructive from central respiratory events, though comprehensive correlation with esophageal pressure remains insufficiently characterized [51, 52, 55]. Similar to RIP, sensor location can potentially influence signal characteristics. Sensors placed high on the upper thorax near the sternal notch may be exposed to smaller thoracoabdominal excursions than those positioned closer to the abdomen that are exposed to larger excursions [27, 32, 51]. It is also imperative to consider that signal reliability exhibits position-dependent variability, with supine positioning typically yielding optimal signal quality and prone positioning demonstrating the greatest susceptibility to signal degradation.

Furthermore, sensor placement superior to the sternal notch on the anterior tracheal surface enables measurement of tracheo-sternal motion, representing an alternative respiratory effort surrogate employing comparable biomechanical principles. This configuration captures the tracheal movements in the craniocaudal and anterior–posterior directions during respiratory cycles, providing an additional metric of inspiratory effort [32].

#### Mandibular motion

Mandibular displacement patterns exhibit temporal synchrony with thoracic motion during respiration, establishing their utility as surrogate markers of respiratory effort. The biomechanical mechanism underlying mandibular motion involves caudal traction forces generated by diaphragmatic contraction, which are transmitted through the trachea to the hyoid apparatus and subsequently to the mandible via submental musculature [56]. Concurrent recruitment of trigeminal motoneurons innervating mandibular muscles for upper airway stabilization may further contribute to mandibular movements [57]. Various methodologies have been explored to quantify mandibular motion in the context of sleep monitoring. These include utilizing strain gauges attached between the nose and chin [58], leveraging a magnet attached to the mandible to track the magnetic field changes [59, 60], and combining accelerometry with gyroscope sensing on the chin [61]. Validation studies comparing mandibular motion signals against established respiratory effort measurements, including esophageal manometry, pulse transit time analysis, and diaphragmatic EMG, have demonstrated proportional agreement to enable differentiation of central and obstructive events [56, 57, 61–64].

Notably, signal quality exhibits relative immunity to changes in head position, though certain sleep postures that constrain head movement against pillow surfaces could introduce artifacts [61]. Sensors placed on the chin may also require shaving the underlying facial hair during application if present [8]. Beyond event classification, recent investigations have established clinical associations between elevated nocturnal respiratory effort burden (quantified via mandibular motion) and comorbid systemic hypertension and type 2 diabetes [65, 66]. Lastly, the utility of mandibular motion has also been expanded to sleep stage classification utilizing machine learning algorithms [67].

#### Suprasternal pressure

Suprasternal pressure (SSP) quantifies respiratory movement by detecting pressure changes at the suprasternal notch, which reflect the dynamic fluctuations in intrathoracic pressure generated by respiratory effort [26, 35]. The measurement principle, initially conceptualized in 1969 and implemented clinically in the 1980 s, leverages the concept that membrane deflection over an occluded volume can function as a surrogate of the pressure variations within [68, 69]. SSP can be acquired via employment of a stethoscope-like sensor housing a coupled acoustic and pressure transducer inside an airtight chamber adherent to the skin surface. SSP is captured by filtering the signal to isolate lower sub-audible pressure oscillations (0.2–20 Hz) corresponding to respiratory effort-induced pressure variations [26]. During inspiration, the deflection of the skin over the sternal notch towards the trachea reduces chamber pressure, while expiration restores baseline pressure. SSP waveform morphology demonstrates proportionality to esophageal pressure both in frequency and amplitude, allowing discrimination between central, mixed, and obstructive events [26, 33, 35, 70, 71].

A comparative study indicated that SSP exhibited superior sensitivity to respiratory effort relative to thoracoabdominal strain gauges, making it less likely to misclassify obstructive events as central [33]. Analogous to tracheal sound sensors, optimal signal acquisition necessitates an airtight sensor-skin interface. Excessive cervical skin laxity may preclude adequate seal formation, preventing sensor utilization in affected individuals [33].

#### Respiratory-induced pulse wave modulation

Photoplethysmography (PPG) represents a non-invasive optical technique that measures blood volume fluctuations in microvascular tissue beds [72]. The widespread adoption of PPG in consumer and medical-grade wearable devices reflects its technical simplicity, economic feasibility, and measurement versatility for cardiovascular and sleep monitoring applications [8, 73]. Respiratory activities manifest within the PPG signal through characteristic modulations of pulse wave amplitude (PWA), signal intensity, and pulse rate variations [74]. Signal processing with bandpass filtering to isolate respiratory frequency components (typically 0.1–0.5 Hz, corresponding to 6–30 breaths per minute) yields waveforms that approximate respiratory patterns in both frequency and amplitude domains [75–77]. This pulse wave modulation arises from rhythmic changes in tissue perfusion at the PPG sensing site throughout the ventilatory cycle. During inspiration, a drop in intrathoracic pressure diminishes stroke volume and reduces vascular perfusion in cutaneous vascular beds. These hemodynamic changes manifest as decreases in PWA and PPG signal intensity, accompanied by compensatory increases in heart rate. Expiration results in the opposite, increasing in PWA and PPG signal intensity while decreasing heart rate [78, 79]. This rhythmic cyclic pattern ceases during central apneas, facilitating differentiation from obstructive events [29, 49, 51, 53, 80–83].

Respiratory-induced pulse wave modulation can be detected at various anatomical sites, including finger/fingertip, chest wall, forehead, earlobe, and ear canal. These characteristics demonstrate site-dependent variation, with central locations typically exhibiting stronger respiratory modulation due to cardiac proximity and reduced interference from sympathetically mediated peripheral vasoconstriction [84]. Chest-wall placement particularly amplifies the respiratory effort signal as cyclic tension over the sternum stretches the skin under the sensor [85].

The systemic nature of cardiovascular-respiratory coupling also confers relative resistance to postural changes during measurement [75]. However, as an optical measurement modality, poor contact, skin pigmentation, skin thickness, hair, tattoo, nail polish, and motion artifacts can affect signal quality and should be considered as confounding variables, necessitating careful consideration in clinical implementation [78]. Pairing PWA-based respiratory assessment with other respiratory effort monitoring methods may potentially enhance robustness and reliability in diagnostic applications.

#### Pulse transit time/pulse arrival time

Pulse transit time (PTT) has been extensively investigated in sleep medicine as a surrogate for respiratory effort and sympathetic activation during respiratory event termination (see the “Pulse transit time/pulse arrival time” section) [86–90]. PTT quantifies the time required for pulse pressure wave propagation between two distinct points within the arterial vasculature. Interest in such temporal intervals emerged in the 1920 s based on their inverse relationship with arterial blood pressure (pulse wave velocity accelerates through stiffer arteries, reducing PTT) [91].

During inspiration, negative intrathoracic pressure increases venous return to the right heart and decreases left ventricular filling, hence decreasing arterial blood pressure and prolonging PTT. The cycle reverses during expiration as increased intrathoracic pressure elevates blood pressure and shortens PTT [92]. PTT correlates well with esophageal pressure in both respiratory rate and magnitude of respiratory effort, enabling discrimination between obstructive and central events [87, 90, 92–95]. As a systemic physiological response, changes in PTT can be assessed between the heart and various peripheral arterial sites such as the finger, toe, earlobe, chest, carotid artery, or femoral artery. For convenience, the most prevalent implementation in sleep medicine measures PTT as the time interval between electrical activation of the ventricles (i.e., R-wave on ECG) and the arrival of the PPG pulse wave at the peripheral location, requiring two independent sensor modalities. This conventional method is more accurately described as *pulse arrival time* because it inherently incorporates the pre-ejection period (PEP), the electromechanical delay between ventricular depolarization and opening of the aortic valve [91]. In most clinical scenarios, including PEP alongside true PTT may not matter because they typically demonstrate concordant directional changes [86]. However, emerging technologies that measure aortic valve opening like seismocardiography may provide enhanced measurement precision [52, 53].

Notably, PTT demonstrates susceptibility to multiple physiological and pathological influences, encompassing autonomic tone fluctuations, cardiac dysfunction, and arterial wall compliance changes with aging. Cardiac arrhythmias and conditions influencing R-wave detection and cardiac contractility may compromise its accuracy [39, 93]. Furthermore, while PTT-based effort assessment generally remains robust across body positions, its reliability may diminish during REM sleep due to autonomic instability [95]. Consequently, PTT optimal utility likely resides within multimodal physiological assessment frameworks rather than as a standalone measure [93].

### Sympathetic activation assessment in sleep-disordered breathing

Sympathetic activation has been recognized as a fundamental physiological response to respiratory events during sleep since the 1970 s [96–98]. During a typical apneic event, hypoxemia and a transient drop in blood pressure trigger the chemoreflex and baroreflex to progressively increase sympathetically activated peripheral vasoconstriction to restore perfusion and blood pressure. Despite a high sympathetic tone, heart rate counterintuitively decreases during respiratory events because of a dominant counteracting parasympathetic influence, a protective mechanism that optimizes pulmonary gas exchange in the absence of ventilation. Upon event termination, resumption of breathing releases parasympathetic inhibition, unmasking accumulated sympathetic drive. These pathophysiological events manifest as “autonomic arousals”, characterized by transient, sudden tachycardia, blood pressure surge, and intensified peripheral vasoconstriction [99–106]. Such autonomic arousals often, though not invariably, coincide with cortical arousals, which require electroencephalography (EEG) for detection [107–109].

Beyond chronic intermittent hypoxemia observed during respiratory events, repeated cycles of sympathetic activation are increasingly linked to cardiovascular diseases [39, 110, 111]. Furthermore, sympathetic activity exhibits distinct patterns during REM sleep, which can aid sleep stage identification, while its increase during periodic limb movements in sleep can potentially confound SDB assessments [112].

#### Microneurography

Microneurography constitutes the reference standard to assess sympathetic activation in SDB by enabling direct quantification of peripheral vasoconstrictor nerve traffic to skeletal muscle vascular beds [92, 113–115]. First developed in the 1960 s and later applied to SDB research in the 1980 s, microneurography records muscle sympathetic nerve activity (MSNA) by inserting a tungsten microelectrode percutaneously into an accessible peripheral nerve [98, 100, 116, 117]. The peroneal nerve at the fibular head represents the most common recording site, though tibial, femoral, median, ulnar, and radial nerves have also been utilized. MSNA typically demonstrates progressive augmentation throughout apneic events, particularly during prolonged episodes, with a pronounced surge at event termination. This surge precedes the vasoconstriction-mediated rise in blood pressure and often coincides with EEG arousal [99, 113, 118]. Elevated MSNA persists during wakefulness in patients with SDB, exhibiting moderate correlation with the apnea–hypopnea index (AHI) [100, 114]. However, due to its invasive nature, microneurography has been confined to research settings, precluding routine clinical application. Reported complication rates have ranged from 0.3% to 9%, primarily localized tenderness at the electrode insertion site [117].

#### Cardiac rate response

The cardiac rate response to apneic events represents perhaps the most recognizable marker of sympathetic activation in response to SDB. First described in the 1980 s, the characteristic “cyclic bradycardia-tachycardia” pattern associated with respiratory events reflects the autonomically mediated response to upper airway occlusion: dominated initially by parasympathetic activation during the event, followed by an abrupt sympathetic surge at event termination [97, 102]. When multiple respiratory events occur sequentially, the repetitive heart rate variation effectively introduces a low-frequency oscillation component corresponding to respiratory event periodicity. This pattern is readily apparent on tachograms, heart rate variability (HRV) plots, and in frequency domain representations [119].

While ECG-derived heart rate remains the benchmark reference, cardiac rate fluctuations and HRV metrics can be captured or derived from other physiological signals, including PPG, EEG, EMG, and esophageal pressure waveforms. Multiple factors, including arrhythmias such as atrial fibrillation and medications like beta-blockers, calcium channel blockers, antiarrhythmic agents, vasoactive and psychoactive medications, can potentially influence autonomic modulation of heart rate and SDB detection sensitivity and specificity [9]. Notably, the performance can be improved by coupling cardiac rate with other complementary signals relevant to SDB, such as oxygen desaturations [120, 121], PWA attenuation, or respiratory pattern analysis (see the “Cardiopulmonary coupling” section). Beyond detecting respiratory events, an impaired or elevated sleep apnea-specific cardiac rate response has also been associated with increased cardiovascular risk [122, 123].

#### Cardiopulmonary coupling

Cardiopulmonary coupling (CPC) integrates the variability in heart rate response to SDB events (see the “Cardiac rate response” section) and respiratory rate variability that is already conveniently embedded within the cardiac signal itself, typically acquired via ECG or PPG. ECG-derived respiration (EDR) can be estimated through transthoracic impedance changes, as ECG electrode positions vary relative to cardiac structures during ventilatory cycles [124]. PPG-derived respiration was previously discussed in the “Respiratory-induced pulse wave modulation” section. Theoretically, other respiratory effort methods from the “Respiratory effort assessment in sleep-disordered breathing” section could also be utilized. CPC exploits the observation that respiratory perturbations often occur at similar intervals as heart rate fluctuations during sequential SDB events, such that detection performance enhances when both demonstrate temporal coupling [125–127]. Notably, low-frequency coupling (LFC) within the 0.01–0.1 Hz band (i.e., 10–100 s periodicities) exhibits a specific association with SDB. Additionally, autonomic modulation of CPC across LFC, very low frequency, and high frequency bands has been utilized in sleep stage classification and has shown potential use in detecting central respiratory events [126, 128].

#### Peripheral vasoconstriction

During most SDB events, sympathetically mediated peripheral vasoconstriction reduces pulsatile blood volume within cutaneous vascular beds. These hemodynamic changes can be quantified using plethysmography at a peripheral site as attenuations in pulse wave amplitude (PWA). First observed in the 1940 s, PWA attenuation during SDB frequently demonstrates temporal association with other hallmarks of respiratory events, including oxygen desaturation, accelerated heart rate, and arousal responses [129–134]. PWA attenuation has been measured at multiple anatomical locations that demonstrate sufficient sympathetic vasoconstriction responsiveness during SDB events. The fingertip generally exhibits the most robust vasoconstrictor response and offers convenient accessibility for sensor placement. In contrast, forehead, ear lobe, and ear canal sites demonstrate comparatively limited vasoconstrictor responses, potentially reducing measurement sensitivity [129, 132, 135–140].

Various plethysmography techniques have been developed to measure peripheral vasoconstriction via PWA assessment, including strain gauges [132], thermography [132], non-contact optical imaging [141], and air displacement apparatuses [142], but PPG is by far the most predominant contemporary technique for peripheral vasoconstriction assessment [52, 143]. Notably, peripheral arterial tonometry (PAT) constitutes a specialized PPG-based implementation that has been widely utilized in sleep medicine since the early 2000s. This technique typically employs a pneumo-optic PAT probe that applies controlled sub-diastolic pressure at the fingertip, effectively mitigating venous pooling and motion artifact and better isolating arterial pulsatile contributions [133, 144].

As a downstream consequence of sympathetic activation, peripheral vasoconstriction demonstrates susceptibility to unrelated non-respiratory physiological stimuli, including environmental noise, periodic limb movements, sleep fragmentation, and poor perfusion from cold exposure or impaired vascular function, which may confound interpretation of SDB severity [92, 123, 145–148]. Excluding PWA attenuation events that are not accompanied by concurrent heart rate acceleration and oxygen desaturation has been shown to increase specificity to detect AHI-defined SDB [149]. Beyond detecting SDB events, specific PWA characteristics have also been associated with a higher risk of incident cardiovascular disease and have been leveraged for sleep stage differentiation algorithms [123, 146, 150, 151].

#### Pulse transit time/pulse arrival time

In addition to its established utility as a respiratory effort surrogate (see the “Pulse transit time/pulse arrival time” section), PTT and pulse arrival time can serve as indirect markers of sympathetic activation at respiratory event termination, to the extent that they reflect blood pressure dynamics. Sympathetically mediated increases in blood pressure elevate vascular tone, stiffen arterial walls, and abruptly shorten PTT [93, 152]. Such transient decreases in PTT are frequently characterized as a sensitive technique to detect autonomic arousals, demonstrating favorable concordance with both AHI and respiratory disturbance index (RDI) [92, 153–158]. However, analogous to PWA as a surrogate of sympathetically mediated vasoconstriction, PTT as a surrogate of sympathetically mediated blood pressure dynamics may have poor specificity to detect arousals. Comparative studies in pediatric populations suggest that PTT exhibits superior specificity relative to PWA, though both modalities may need to be used in conjunction with other confirmatory signals [145, 159]. Additionally, baseline PTT and the magnitude of PTT response to SDB events have been associated with SDB severity and markers of subclinical cardiovascular disease, including left ventricular mass, carotid plaque, and coronary artery calcification [160, 161].

## Discussion

Early portable monitoring devices primarily relied on conventional respiratory channels borrowed from in-lab PSG, such as thermistor- or nasal cannula-based airflow detection and respiratory effort assessment using RIP [9]. The subsequent decade and a half has witnessed unprecedented progress in sensor miniaturization and digital signal processing techniques that have spurred the proliferation of numerous novel HSAT platforms. Contemporary devices increasingly incorporate sophisticated physiological measurement methodologies formerly confined to research settings. Most of these emerging technologies have been extensively reviewed in a recent device-focused review article [8].

Despite substantial technological innovation and their increasing clinical adoption, many respiratory signals, such as tracheal sounds-derived acoustic flow, mandibular movements, suprasternal pressure, PPG pulse wave amplitude, respiratory-induced pulse wave modulation, pulse transit time/pulse arrival time, have received minimal recognition in clinical practice guidelines. In addition, HSAT classification frameworks have remained essentially static, lacking the iterative refinement necessary to maintain alignment with three decades of clinical evolution. For instance, the traditional AASM type I–IV Portable Monitoring taxonomy predominantly emphasizes airflow and respiratory effort metrics, whereas an alternative classification system in the United States utilized for coding purposes only references examples of airflow and sympathetic activation measures (under Current Procedural Terminology® code 95800) [9, 10, 12]. The SCOPER (Sleep, Cardiovascular, Oximetry, Position, Effort, Respiratory) HSAT categorization, based on 6 measured physiological metrics, represents an innovative approach offering more granular device characterization. However, its clinical implementation has been limited due to operational complexity and the practical challenges of applying multidimensional classification in routine clinical workflows [10].

PAT technology, which quantifies sympathetic nervous system activation, exemplifies this difficulty. Although PAT achieved broader acceptance in the late 2000 s, its integration into the established HSAT classification schemas proved challenging, with inconsistent categorization as a respiratory parameter, cardiac parameter, or simply handled as an exception requiring special consideration [3, 10, 12, 162]. Such ambiguity was acknowledged at the time, illustrating the historical difficulty of integrating emerging tools/methodologies into established diagnostic framework [10].

This narrative review specifically examined respiratory signal analysis methodologies and underlying physiological mechanisms. The respiratory signals were stratified into three distinct physiological domains: airflow dynamics, respiratory effort mechanics, and sympathetic activation patterns, each demonstrating unique response profiles during respiratory events. Explicitly bringing these physiological domains into a future classification framework may enhance conceptual clarity regarding device mechanistic principles and functional capabilities compared to existing classification systems. Table 4 presents a comprehensive analysis of measurement methodologies utilized for interrogation of these three respiratory signal domains across multiple modalities and diagnostic platforms: in-lab PSG, legacy flow-based HSAT devices, and emerging HSAT technologies.

**Table 4:** Respiratory analysis signal domains and measurement technologies utilized by various sleep testing methods and devices

Signal domain	Measurement technologies	PSG	Flow-Based HSAT Devices	Emerging HSAT device examples
Airflow	*Pneumotachograph*	○		
	Nasal pressure transducer	●	●	
	Thermistor	●	●	Sunrise
	Thoracoabdominal effort-derived flow	●	◑	Wesper Lab
	Tracheal sounds			AcuPebble, BresoDX1
Respiratory effort	*Esophageal pressure*	○		
	Chest wall electromyography	○		
	Respiratory inductance plethysmography	●	●	
	Chest/tracheosternal movement			ANNE Sleep, BresoDX1, SANSA, WatchPAT
	Mandibular motion			Sunrise
	Suprasternal pressure			AcuPebble
	Respiratory-Induced Pulse Wave Modulation	◑	◑	SANSA, SleepImage
	Pulse transit time/Pulse arrival time	●	○	ANNE Sleep, SANSA
Sympathetic activation	*Microneurography*			
	Cardiac rate response	●	●	PPG- and ECG-based devices
	Cardiopulmonary coupling	◑	◑	SANSA, SleepImage
	Peripheral vasoconstriction	●	◑	ANNE Sleep, Belun Ring, NightOwl, SleepImage, Somfit, TipTraQ, WatchPAT
	Pulse transit time/Pulse arrival time	●	○	ANNE Sleep, SANSA

For PSG and conventional flow-based HSAT columns, filled circles (●) represent technologies typically found in a standard montage, half-filled circles (◑) denote technologies that can be derived from a standard montage, and empty circles (○) indicate technologies that are rarely applied. *Italics* are used to highlight the gold standard methodology for each signal domain. Technologies in the emerging HSATs section were defined based on information from peer-reviewed publications and regulatory documents, along with confirmation from manufacturers

*ECG*, electrocardiography; *HSAT*, home sleep apnea test; *PPG*, photoplethysmography; *PSG*, polysomnography

Inspection of Table 4 reflects the inherent trade-off between diagnostic precision and practical feasibility in respiratory monitoring. Notably, even within in-lab polysomnography, gold standard methods of measuring each respiratory analysis domain are rarely implemented in routine clinical practice. Surrogate measurement techniques introduced during the pre-HSAT era in the context of polysomnography benefit from extensive temporal validation and clinician familiarity. However, these methods remain physiological approximations, nevertheless. As novel surrogate methods transition from investigational contexts to clinical deployment, they must undergo rigorous performance evaluation to characterize their specific operational strengths and weaknesses and ensure clinical integrity. Insights derived from these newer approaches may even find leverage and offer translational value in PSG applications in addition to HSAT, potentially enhancing the identification and discrimination of SDB events. Broader integration of these complementary measurement methodologies across physiological signal domains may further facilitate standardization in respiratory event detection and SDB diagnosis compared to existing, often subjective, criteria derived from limited signals (e.g., percentage airflow amplitude reductions relatively to a visually determined baseline).

This methodological review also highlights the necessity of multimodal signal integration for accurate SDB event identification, as no single measure is sufficient in isolation. For example, sympathetic activation methodologies alone may have limited specificity for event detection and inadequate discriminatory capacity to distinguish central from obstructive events. Similarly, airflow measurements in isolation provide insufficient information for central apnea discernment, while respiratory effort recognition alone may fail to adequately recognize obstructive events. Respiratory signals must therefore be synthesized alongside other sleep study parameters to optimize SDB event detection [31]. In practice, respiratory signal amplitude and morphology are also influenced by specific sensor technologies, device designs and specifications, calibration, filtering, and other signal processing parameters. These factors should be understood as they can potentially impact the implementation of rigid scoring criteria. Collectively, a comprehensive evaluation of home SDB diagnostics necessitates thoughtful integration of multiple well-characterized respiratory analysis signals with pulse oximetry, sleep stages, body position, and other contextual signals (e.g., snoring and actigraphy).

## Conclusion

The convergence of cutting-edge sleep technologies, sensor miniaturization, advanced signal processing, and artificial intelligence/machine learning has profoundly reshaped the landscape of the SDB diagnostic paradigm. Contemporary HSAT platforms have already incorporated physiological measurement methodologies that substantially diverge from conventional technologies, yet our prevailing classification frameworks remain anchored to decades-old taxonomic structures that fail to accommodate these innovations. Looking ahead, the trajectory of SDB monitoring will likely encompass novel methodologies, including ear-worn sensors (earables), non-contact radar-based sensors (airables), ballistocardiography-based under-mattress nearable sensors, and applications leveraging smartphones or smartwatches [163].

We contend that a comprehensive revision of sleep testing classification systems constitutes a pressing imperative for the sleep medicine community. Formulating a classification schema that balances both conceptual comprehensiveness and operational practicality presents considerable challenges. We advocate for a reconceptualization of sleep testing taxonomies to ensure clinical relevance and diagnostic utility amid accelerating technological evolution. Prospective classification frameworks for SDB diagnostic tools (and longitudinal monitoring platforms) should maintain sufficient architectural adaptability to integrate emerging technologies without requiring complete restructuring with each innovation.

## Data Availability

Not applicable.
